# BER Performance Analysis of Non-Coherent *Q*-Ary Pulse Position Modulation Receivers on AWGN Channel

**DOI:** 10.3390/s21186102

**Published:** 2021-09-12

**Authors:** Xianhua Shi, Yimao Sun, Jie Tian, Maolin Chen, Youjiang Liu, Nan Xie, Jian Zhang

**Affiliations:** 1 Institute of Electronic Engineering, CAEP, Mianyang 621999, China; tianjie@caep.cn (J.T.); maolin66@126.com (M.C.); liuyj04@163.com (Y.L.); xienanmail@126.com (N.X.); 2 College of Computer Science, Sichuan University, Chengdu 610065, China; yimaosun@scu.edu.cn; 3 School of Electronic Science and Engineering, University of Electronic Science and Technology of China, Chengdu 611731, China; jianzhang@uestc.edu.cn

**Keywords:** *Q*-ary, pulse position modulation, AWGN channel, non-coherent, bit error rate

## Abstract

This paper introduces the structure of a *Q*-ary pulse position modulation (PPM) signal and presents a noncoherent suboptimal receiver and a noncoherent optimal receiver. Aiming at addressing the lack of an accurate theoretical formula of the bit error rate (BER) of a *Q*-ary PPM receiver in the additive white Gaussian noise (AWGN) channel in the existing literature, the theoretical formulas of the BER of a noncoherent suboptimal receiver and noncoherent optimal receiver are derived, respectively. The simulation results verify the correctness of the theoretical formulas. The theoretical formulas can be applied to a *Q*-ary PPM system including binary PPM. In addition, the analysis shows that the larger the *Q*, the better the error performance of the receiver and that the error performance of the optimal receiver is about 2 dB better than that of the suboptimal receiver. The relationship between the threshold coefficient of the suboptimal receiver and the error performance is also given.

## 1. Introduction

Pulse position modulation (PPM) technology is a discrete pulse communication technology that is different from continuous wave communication technology. PPM transmits information by transmitting discrete pulse signals in the time domain. The position of pulse signals is determined by the data to be transmitted. The PPM signal has the advantages of a low duty cycle, high peak power and high energy efficiency. It has been widely studied in the field of optical communication [1,2,3], underwater communication [4,5] and radio communication [6,7,8]. Early PPMs belong to analog modulation technology. The data to be transmitted are continuously variable analog data, and the position of the pulse signal is also continuously variable in the time domain. The current PPM technology is usually digital modulation, and the data to be transmitted are also digital signals. Each digital PPM symbol carries *M*-bit data, and the symbol duration $T_{s}$ is evenly divided into $Q=2^{M}$ time slots. One of the *Q* time slots is selected according to the *M*-bit data to be transmitted to transmit a pulse signal. Therefore, the pulse signal can only appear in a limited time slot position and is not continuously variable. A digital PPM in which each symbol duration is divided into *Q* uniform time slots is commonly referred to as *Q*-ary PPM [9,10]. In particular, when the PPM symbol carries only one bit of data, the number of time slots *Q* is equal to 2. At this time, we call this binary PPM. The PPM technologies studied in this paper refer to digital modulation PPM technology.

PPM modulation technology has been widely used in the field of optical communication, such as optical fiber communication and deep space communication, and its performance has also been studied, including the binary PPM modulation system and *Q*-ary PPM modulation system. The research shows that PPM modulation technology is an efficient optical communication technology with high energy efficiency and strong anti-interference ability.

PPM modulation technology has also been widely used in the field of wireless communication. In particular, after the FCC licensed the 3–10 GHz band to ultra wideband (UWB) technology for free, UWB communication technology has been studied a great deal. PPM, as a technology to realize UWB communication, has also been studied to a great extent [11,12], including regarding its bit error performance [13,14,15,16,17,18]. However, most of the literature on wireless communication has studied the binary PPM system, and only a small amount of the literature has focused on the *Q*-ary PPM system [19,20]. To the best of our knowledge, there is no accurate description of the bit error performance of the *Q*-ary PPM system under the AWGN channel. There are many studies on the bit error performance of the *Q*-ary PPM system in the field of optical communication, but most of them are based on the Poisson channel. These results are not applicable to the scenario of the AWGN channel [3,21,22].

As the noncoherent PPM system has the advantages of simple implementation, we mainly study the noncoherent PPM system and introduce the suboptimal receiver and the optimal receiver of the noncoherent *Q*-ary PPM system, respectively. The suboptimal receiver makes decisions through the threshold decision method. This method has low complexity, and the optimal receiver adopts the method of selecting the largest decision that has a better performance. We derive the accurate BER performance expression of the suboptimal receiver and the accurate BER performance expression of the optimal receiver for a noncoherent *Q*-ary PPM system and carry out a numerical simulation. The simulation results are in good agreement with the theoretical expression, which shows the correctness of the theoretical formula. We also analyze the change in the bit error performance of a noncoherent *Q*-ary PPM system with the increase in *Q*. The results show that the bit error performance is improved with the increase in *Q*; that is, less energy is required to transmit one bit of information. Therefore, a PPM system with a larger *Q* can be selected in some application scenarios that require higher energy efficiency. In addition, we also compare the performance of the suboptimal receiver and the optimal receiver in a noncoherent PPM system. The results show that the performance of the optimal receiver is about 2 dB better than that of the suboptimal receiver, but the implementation complexity of the suboptimal receiver is low, so it should be considered comprehensively for use.

The rest of this paper is organized as follows. Section 2 introduces the PPM signal structure and describes the noncoherent PPM suboptimal receiver and the optimal receiver, respectively. In Section 3, the accurate expressions of the error performance of the noncoherent PPM suboptimal receiver and the optimal receiver in the Gaussian channel are derived, respectively, and the influence of the decision threshold of the noncoherent PPM suboptimal receiver on the error performance is analyzed. In Section 4, the suboptimal error performance and the best error performance of the noncoherent PPM receiver are numerically simulated, and the simulation results are compared with the theoretical expressions derived in this paper. Section 5 summarizes this paper.

## 2. System Model

In this section, the PPM signal structure is introduced first. Then, a suboptimal noncoherent PPM receiver is developed. Finally, we present the optimal noncoherent PPM receiver.

### 2.1. Structure of PPM Signal

**Figure 1:**
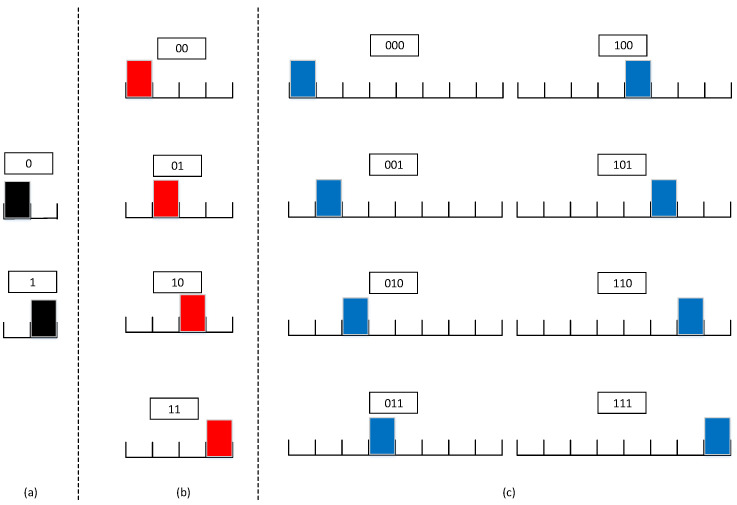
*Q*-ary PPM signal. (**a**) is a binary PPM, (**b**) is a 4-ary PPM and (**c**) is a 8-ary PPM.

PPM is a communication technology that transmits signals through different positions of pulse signals in the time domain. It can be divided into a binary PPM signal and *Q*-ary PPM signal. The binary PPM signal can also be regarded as a special case of the *Q*-ary PPM signal. Assuming that the duration of a PPM symbol is $T_{s}$, $T_{s}$ is evenly divided into $Q=2^{M}$ equal time slots and a time slot is selected from the *Q* time slots to transmit a pulse signal according to the data to be transmitted. For the convenience of analysis, we assume that the shape of the transmitted pulse is rectangular, and the pulse duration is equal to the time slot width. The signal model is

(1) $$s\left(t\right)=A\sum_{j}p(t-jT_{s}-k\delta )\;.$$

where p(t) represents the pulse waveform, $T_{s}$ is the PPM symbol duration and *k* is a vector composed of data to be transmitted, where $k\in [1,2^{M}]$. δ indicates the slot width. The PPM signal is shown in Figure 1, where (a) is a binary PPM, (b) is a 4-ary PPM and (c) is a 8-ary PPM. The mapping of the data in the figure to PPM symbols adopts the natural mapping method.

### 2.2. Suboptimal Noncoherent PPM Receiver

**Figure 2:**
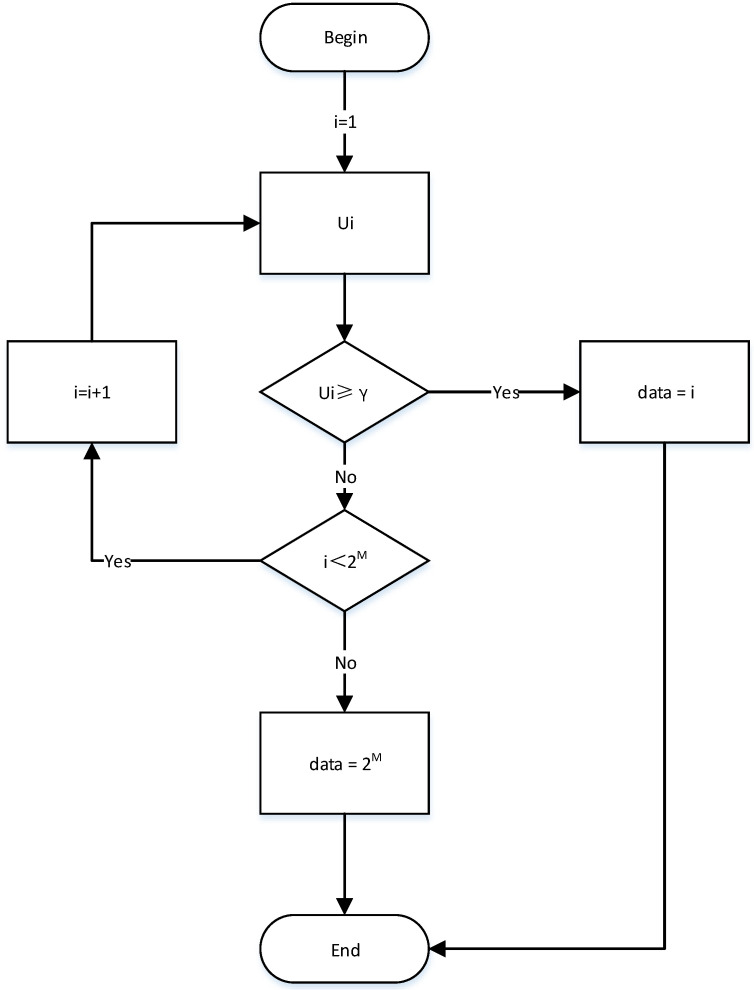
Symbol decision flow of a suboptimal receiver.

The suboptimal PPM receiver first recovers the pulse signal through envelope detection and then determines the received information by judging which of the $2^{M}$ time slots the pulse signal is in. In order to determine which time slot the PPM pulse is in, the suboptimal PPM receiver first needs to complete time synchronization; that is, it needs to determine the boundary of PPM symbols, usually including a transmission reference synchronization method, side-by-side synchronization method and full blind synchronization method [23,24]. This paper does not study the synchronization method, and the subsequent analysis assumes that the system has been synchronized well. For the synchronized system, the decision flow of the suboptimal PPM receiver is shown in Figure 2.

1. Start symbol decision;
2. Envelope value sampling starts from time slot i=1;
3. Compare the sampling value $U_{i}$ with the threshold value γ;
4. If the sampling value exceeds the threshold value, the pulse is considered to be in the first time slot, the information represented by the first time slot is output and the current symbol decision ends;
5. If the sampling value is less than the threshold value, it is considered that the pulse is not in this time slot, and the envelope value of the next time slot is sampled and compared with the threshold value;
6. This continues until the sampling value of the $k\in [1,2^{M}]$ time slot exceeds the threshold value, the information represented by the *k*th time slot is output and the current symbol decision is ended;
7. In particular, if the sampling values from slot 1 to slot $2^{M}$ are less than the threshold value, we agree that this symbol outputs the information represented by slot $2^{M}$ and end the current decision.

### 2.3. Optimal Noncoherent PPM Receiver

**Figure 3:**
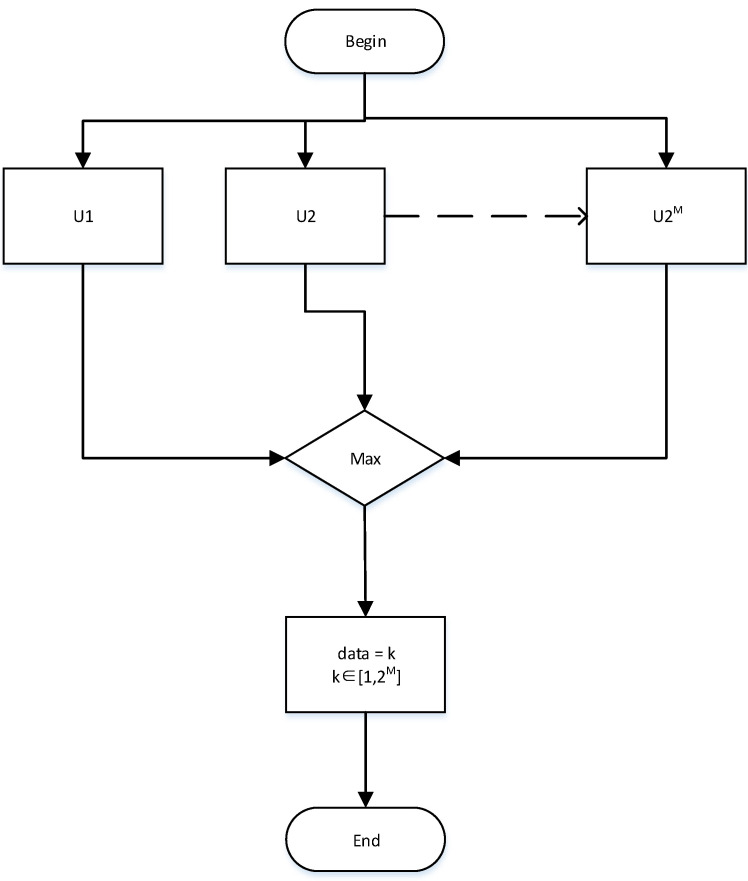
Symbol decision flow of optimal receiver.

The optimal PPM receiver also recovers the pulse signal through envelope detection and then determines the received information by judging which of the $2^{M}$ time slots the pulse signal is in. The decision process is shown in Figure 3.

1. Start symbol decision;
2. Sample the envelope values of time slots $1,2,\ldots 2^{M}$;
3. Select the maximum time slot $U_{k}$ in the sampling values $U_{1}$, $U_{2}$, ⋯, $U_{2^{M}}$; determine the time slot where the signal is located; and output data *k*;
4. End decision.

## 3. Performance Analysis

The PPM system has two demodulation methods: the suboptimal demodulation method and optimal demodulation method. The bit error performance of the two demodulation methods is analyzed below. In order to facilitate the analysis and without losing generality, we make the following assumptions in the subsequent analysis: the system has been synchronized well and the pulse signal is uniformly distributed in the possible time slot. According to the analysis in Section 2, after receiving the signal, the suboptimal PPM receiver and the optimal PPM receiver first recover the pulse signal through envelope detection. The noise envelope sampling without a signal follows the Rayleigh distribution,

(2) $$p\left(u_{N}\right)=\frac{u_{N}}{\sigma^{2}}\exp\left(-\frac{u_{N}^{2}}{\sigma^{2}}\right)\;,$$

and the envelope sampling with a signal follows the Rician distribution,

(3) $$p\left(u_{s}\right)=\frac{u_{S}}{\sigma^{2}}\exp\left(-\frac{u_{S}^{2}+A^{2}}{2\sigma^{2}}\right)I_{0}\left(\frac{Au_{S}}{\sigma^{2}}\right)\;.$$

where $u_{N}$ is the noise slot envelope sampling value, $u_{S}$ is the signal pulse slot envelope sampling value, $\sigma^{2}$ is the covariance of white Gaussian noise, *A* is the amplitude of the pulse signal and $I_{0}\left(\cdot \right)$ is the zero-order Bessel function.

### 3.1. Performance of Suboptimal Receiver

Each pulse of the PPM system carries *M*-bit information, and each symbol is divided into $Q=2^{M}$ time slots. The basic idea of the demodulation of a suboptimal PPM system is as follows: first, set a threshold value, start from the first time slot of each symbol and successively compare the envelope sampling value of each time slot with the threshold value. When the sampling value of a time slot envelope exceeds the threshold for the first time, it can be considered that the corresponding pulse signal is transmitted in the slot at this time, and it can be determined as the corresponding *M*-bit information; in particular, if the sampling values from the first time slot to the $2^{M}$ time slot do not exceed the threshold, we agree to determine that the $2^{M}$ time slot has sent a signal.

Based on the above idea, assuming that the transmitted signal pulse is located in the *k*th time slot, if the sampling values of the first k−1 time slots are less than the threshold value but the sampling value of the *k*-th time slot is also less than the threshold value, the PPM symbol judgment is incorrect. We call this error leakage error $p_{1}$. If at least one sampling value in the first k−1 time slot exceeds the threshold, a judgment error also occurs, and this corresponds to a false alarm error $p_{2}$. Adding $p_{1}$ and $p_{2}$ allows for the bit error performance expression of the suboptimal PPM receiver to be obtained. However, when calculating the false alarm error $p_{2}$, it is necessary to consider the case that one sampling value exceeds the threshold value in the first k−1 time slots and that two sampling values exceed the threshold value. Until k−1 sampling values exceed the threshold value, the calculation is quite complex. Therefore, we first calculate the correct probability of the symbol judgment of the suboptimal PPM receiver and obtain the error probability by taking its complement to obtain the bit error performance expression of the suboptimal PPM receiver.

Based on the above considerations, assuming that the signal pulse transmitted by the PPM system is located in the *k*th time slot, the sampling envelope value of the first k−1 time slots does not exceed the threshold, and when the envelope sampling value of the *k*th time slot exceeds the threshold, the transmitted symbol decision is correct. It should be noted that, when the transmitted signal is the $2^{M}$ time slot, as long as the envelope sampling value of the first $2^{M-1}$ time slots does not exceed the threshold, the decision is correct regardless of whether the envelope sampling value of the $2^{M}$ time slot exceeds the threshold.

The above is the case in which a symbolic decision is correct, and the case where the decision is incorrect can be obtained by taking its complement. We first calculate the probability of a correct decision and then obtain the probability of an incorrect decision by taking its complement.

Defining the threshold coefficient as γ, the decision threshold is $U_{0}=\gamma A$. Then, the probability that the noise slot envelope sampling value does not exceed the threshold is

(4) $$P_{c1}=\int_{0}^{U_{0}}p\left(u_{N}\right)du_{N}\;.$$

If the envelope value of each noise time slot is independent and identically distributed, the probability that the envelope value of the first k−1 noise time slots does not exceed the threshold is

(5) $${P_{c1}}^{\prime }\left(k\right)=\prod_{i=1}^{k-1}P_{c1}\;.$$

When the *k*th time slot contains a signal, the probability that its envelope value exceeds the threshold is

(6) $$P_{c2}\left(k\right)=\int_{U_{0}}^{+\infty }p\left(u_{S}\right)du_{S}\;.$$

Therefore, when $k⩽2^{M-1}$, the probability of correct symbol judgment is

(7) $$P_{c3}\left(k\leq 2^{M-1}\right)={P_{c1}}^{\prime }\left(k\right)\cdot P_{c2}\left(k\right)\;.$$

When $k=2^{M}$, the probability of correct symbol judgment is

(8) $$P_{c3}\left(k=2^{M}\right)={P_{c1}}^{\prime }\left(2^{M}\right)\cdot P_{c2}\left(2^{M}\right)+{P_{c1}}^{\prime }\left(2^{M}\right)\cdot \left[1-P_{c2}\left(2^{M}\right)\right]={P_{c1}}^{\prime }\left(2^{M}\right)\;.$$

Since *k* is uniformly distributed in $2^{M}$ time slots, the probability of the correct symbol judgment of the suboptimal receiver of the noncoherent PPM system is

(9) $$P_{c}=\frac{1}{2^{M}}\left(\sum_{k=1}^{2^{M}-1}P_{c3}\left(k\leq 2^{M-1}\right)+P_{c3}\left(k=2^{M}\right)\right)\;.$$

Thus, the symbol error rate is

(10) $$P_{ew}=1-P_{c}\;.$$

Substituting (2)–(9) into (10) yields

(11) $$\begin{array}{cc} P_{ew} & =1-\frac{1}{2^{M}}\left\{\sum_{k=1}^{2^{M}-1}\left[\prod_{i=1}^{k-1}\int_{0}^{U_{0}}\frac{u_{N}}{\sigma^{2}}e^{-\frac{{u_{N}}^{2}}{\sigma^{2}}}du_{N}\right]\cdot \int_{U_{0}}^{+\infty }\frac{u_{S}}{\sigma^{2}}e^{\left(-\frac{{u_{S}}^{2}+A^{2}}{2\sigma^{2}}\right)}I_{0}\left(\frac{Au_{S}}{\sigma^{2}}\right)du_{S}\right.\\ & \;\;\;\left.+\prod_{i=1}^{2^{M}-1}\int_{0}^{U_{0}}\frac{u_{N}}{\sigma^{2}}e^{-\frac{{u_{N}}^{2}}{\sigma^{2}}}du_{N}\right\}\\ \; & =1-\frac{1}{2^{M}}\left\{\sum_{k=1}^{2^{M}-1}\left[{\left(1-e^{-\gamma^{2}\frac{A^{2}}{2\sigma^{2}}}\right)}^{k-1}\cdot Q_{1}\left(\frac{A}{\sigma },\gamma \frac{A}{\sigma }\right)\right]+{\left(1-e^{-\gamma^{2}\frac{A^{2}}{2\sigma^{2}}}\right)}^{2^{M}-1}\right\}\;. \end{array}$$

where $Q_{1}\left(a,b\right)$ is the Marcum-*Q* function. As $A^{2}/2\sigma^{2}=M\cdot E_{b}/N_{0}$, there are

(12) $$\begin{array}{cc} P_{ew} & =1-\frac{1}{2^{M}}\left\{\sum_{k=1}^{2^{M}-1}\left[{\left(1-e^{-\gamma^{2}M\cdot \frac{E_{b}}{N_{0}}}\right)}^{k-1}\cdot Q_{1}\left(\sqrt{2M\cdot \frac{E_{b}}{N_{0}}},\gamma \sqrt{2M\cdot \frac{E_{b}}{N_{0}}}\right)\right]\right.\\ \; & \;\;\;\left.+{\left(1-e^{-\gamma^{2}M\cdot \frac{E_{b}}{N_{0}}}\right)}^{2^{M}-1}\right\}\;. \end{array}$$

According to the assumption that the pulse signal is uniformly distributed in the possible time slots, the conversion relationship between the symbol error rate and bit error rate (BER) is [25]

(13) $$P_{eb}=\frac{2^{M-1}}{2^{M}-1}P_{ew}\;.$$

Hence, the BER of the suboptimal receiver of noncoherent PPM system is

(14) $$\begin{array}{cc} P_{eb} & =\frac{2^{M-1}}{2^{M}-1}\left\{1-\frac{1}{2^{M}}\left\{\sum_{k=1}^{2^{M}-1}\left[{\left(1-e^{-\gamma^{2}M\cdot \frac{E_{b}}{N_{0}}}\right)}^{k-1}\cdot Q_{1}\left(\sqrt{2M\cdot \frac{E_{b}}{N_{0}}},\gamma \sqrt{2M\cdot \frac{E_{b}}{N_{0}}}\right)\right]\right.\right.\\ \; & \;\;\;\left.\left.+{\left(1-e^{-\gamma^{2}M\cdot \frac{E_{b}}{N_{0}}}\right)}^{2^{M}-1}\right\}\right\}\;. \end{array}$$

**Figure 4:**
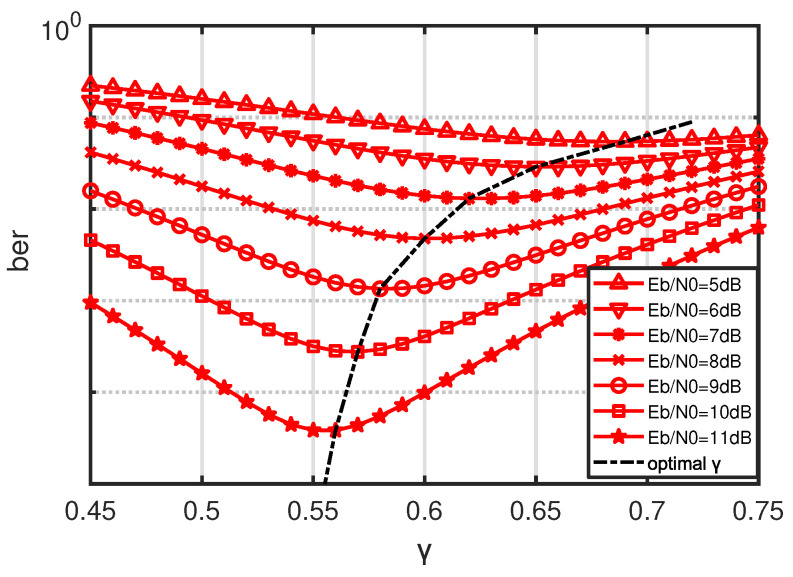
BER of 8-ary noncoherent PPM suboptimal receiver vs. γ.

As can be seen from the above formula, the threshold coefficient γ has a great impact on the performance of the noncoherent PPM with a sub-optimal receiver. The correct selection of the threshold coefficient can lead to better bit error performance. The impact of the threshold coefficient on the bit error performance is shown in Figure 4.

Figure 4 gives the BER performance of the noncoherent suboptimal receiver of the 8-ary PPM versus the value of γ, where the horizontal coordinate is the γ, the vertical coordinate is the BER, the red line indicates the BER at different $E_{b}/N_{0}$ and γ and the black dashed line indicates the trend of the optimal γ with $E_{b}/N_{0}$. It can be seen that the optimal value of γ changes dynamically with $E_{b}/N_{0}$; thus, in order to obtain the best demodulation performance, the value of γ should be adjusted dynamically.

### 3.2. Performance of Optimal Receiver

Each pulse of the PPM system carries *M*-bit information, and each symbol is divided into $Q=2^{M}$ time slots. The basic idea of optimal PPM system demodulation is to successively sample the envelope sampling value of the first time slot to the envelope value of the $2^{M}$ time slot, to compare the size and to judge the time slot with the largest sampling value as the time slot sent by the pulse signal to judge it as the corresponding *M*-bit information. The optimal receiver does not need to set the threshold value, so the difficulty of the dynamic adjustment of the threshold is avoided.

Based on the above idea, assuming that the transmitted pulse signal is in the *k*th time slot, the decision error occurs when the envelope sampling value of one and only one noise time slot exceeds the envelope sampling value of the *k*th time slot. When the envelope sampling value of only two noise time slots exceeds the envelope sampling value of the *k*th time slot, a decision error is also made. By analogy, when the envelope sampling value of all noise time slots exceeds the envelope sampling value of the *k*th time slot, an error is also determined. Only by adding the probabilities of all the above decision error cases can the BER of the optimal noncoherent PPM receiver be obtained, but the calculation is very complex. Therefore, we also calculate the BER of the optimal noncoherent PPM receiver by calculating the probability of a correct decision first and then by finding its complement.

According to the above considerations, it is assumed that the signal pulse transmitted by the PPM system is located in the *k*th time slot. As long as the envelope sampling value of all noise time slots does not exceed the envelope sampling value of the *k*th time slot, the symbol decision is correct. First, the probability that a noise slot envelope sampling value does not exceed the signal pulse slot envelope sampling value is

(15) $$P_{c1}=\int_{0}^{u_{S}}p\left(u_{N}\right)du_{N}\;.$$

Then, the probability that all noise slot envelope sampling values are less than the signal pulse slot envelope sampling values is

(16) $$P_{c2}=\prod_{i=1}^{2^{M}-1}P_{c1}\;.$$

Taking the average of all possible sampling values of the signal yields

(17) $$P_{c3}=\int_{0}^{\infty }P_{c2}\cdot p\left(u_{S}\right)du_{S}\;.$$

Since the signal pulses are evenly distributed in all possible time slots, the probability of a correct decision for any symbol is

(18) $$P_{c}=\frac{1}{2^{M}}\sum_{i=1}^{2^{M}}P_{c3}=P_{c3}\;.$$

By taking its complement, it can be obtained that the optimal symbol error rate of the noncoherent PPM receiver is

(19) $$P_{ew}=1-P_{c}\;.$$

Substituting (2), (3) and (15)–(18) to (19) yields

(20) $$P_{ew}=\sum_{k=1}^{2^{M}-1}\left(\begin{array}{c} 2^{M}-1\\ k \end{array}\right)\frac{{\left(-1\right)}^{k+1}}{k+1}\exp\left(-\frac{k}{k+1}\frac{A^{2}}{2\sigma^{2}}\right)$$

where

(21) $$\left(\begin{array}{c} 2^{M}-1\\ k \end{array}\right)=\frac{\left(2^{M}-1\right)!}{k!\left(2^{M}-1-k\right)!}\;.$$

Considering $A^{2}/2\sigma^{2}=M\cdot E_{b}/N_{0}$, then (20) is simplified as

(22) $$P_{ew}=\sum_{k=1}^{2^{M}-1}\left(\begin{array}{c} 2^{M}-1\\ k \end{array}\right)\frac{{\left(-1\right)}^{k+1}}{k+1}\exp\left(-\frac{k}{k+1}\cdot M\cdot \frac{E_{b}}{N_{0}}\right)\;.$$

Similarly, from the conversion relationship between the symbol error rate and BER (13), the optimal BER of the noncoherent PPM receiver is

(23) $$\begin{array}{cc} P_{eb} & =\frac{2^{M-1}}{2^{M}-1}P_{ew}\\ \; & =\frac{2^{M-1}}{2^{M}-1}\cdot \sum_{k=1}^{2^{M}-1}\left(\begin{array}{c} 2^{M}-1\\ k \end{array}\right)\frac{{\left(-1\right)}^{k+1}}{k+1}\exp\left(-\frac{k}{k+1}\cdot M\cdot \frac{E_{b}}{N_{0}}\right)\;. \end{array}$$

The above BER is consistent with the BER of the noncoherent demodulation of the *M*-ary orthogonal signal because the PPM signal is also an orthogonal signal in essence.

## 4. Simulations

According to the theoretical derivation in Section 3, the BER performance of the noncoherent PPM suboptimal receiver and optimal receiver is numerically simulated in the AWGN channel, and it is assumed that the system has been synchronized well.

### 4.1. BER Performance of Suboptimal Receiver

**Figure 5:**
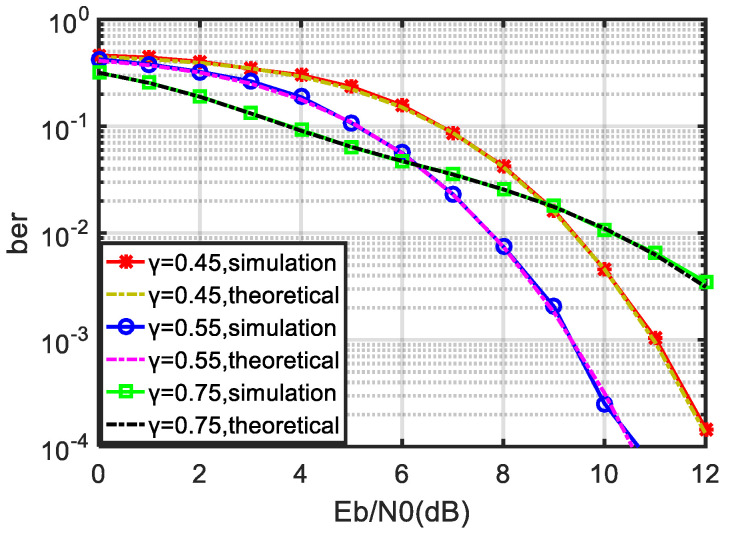
BER performance of noncoherent PPM with different threshold coefficients.

According to the analytical result in Section 3.1, the bit error performance of noncoherent PPM is related to the selection of the threshold coefficient γ, and the optimal coefficient changes dynamically with the change of $E_{b}/N_{0}$. First, we simulate and verify this. For comparison, 8-ary PPM is used in the simulation, and the threshold coefficients are 0.45, 0.55 and 0.75. The value range of $E_{b}/N_{0}$ is 0–12 dB, and the bit error rate is calculated to $10^{-4}$. The simulation results are shown in Figure 5.

It can be seen in Figure 5 that the optimal threshold coefficient changes dynamically with the change in $E_{b}/N_{0}$. When $E_{b}/N_{0}$ is less than 6 dB, the bit error performance is better when the threshold coefficient is 0.75. However, when $E_{b}/N_{0}$ is greater than 6 dB, the bit error performance is better when the threshold coefficient is 0.55. The above simulation verifies the theoretical derivation in Section 3.1. In order to facilitate the analysis, the threshold coefficient is uniformly taken as 0.55 in the rest of this paper.

**Figure 6:**
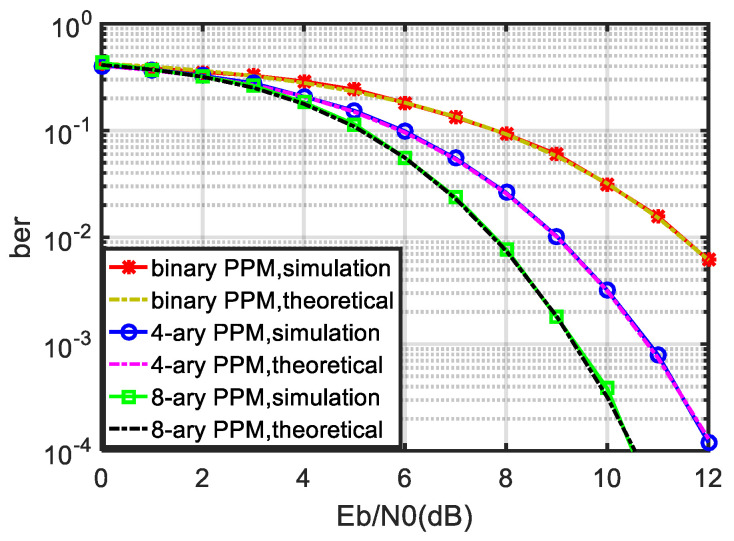
BER performance of noncoherent PPM suboptimal receiver.

Next, the bit error performance of binary PPM, 4-ary PPM and 8-ary PPM is numerically simulated. The threshold coefficient is 0.55, the value range of $E_{b}/N_{0}$ is 0–12 dB and the BER is counted as $10^{-4}$. The simulation results are shown in Figure 6.

In Figure 6, the BER performances of binary PPM, 4-ary PPM and 8-ary PPM suboptimal receivers are evaluated, in which the theoretical curve is from the theoretical formula derived in Section 3.1. The simulation curve is the numerical simulation result. The simulation process first randomly generates the initial binary bit stream A, modulates it into a PPM signal, then sends it to the AWGN channel and then restores it to binary bit stream B through the suboptimal receiver in this paper. We compare the similarities and differences between B and A and calculate the BER to obtain the simulation BER curve.

We can see in Figure 6 that the numerical simulation results are in good agreement with the theoretical formula deduced in Section 3.1, which verifies the correctness of the theoretical formula of the error code of the noncoherent PPM suboptimal receiver deduced in this paper. The theoretical formula is applicable to the *Q*-ary PPM system, including binary PPM. In addition, it can be seen from Figure 6 that the larger the *Q*, the better the bit error performance of the noncoherent PPM suboptimal receiver. This is because the PPM signal essentially corresponds to an orthogonal signal and conforms to the basic characteristics of the orthogonal signal, which further verifies the correctness of the theoretical bit error formula deduced in this paper.

### 4.2. BER Performance of Optimal Receiver

**Figure 7:**
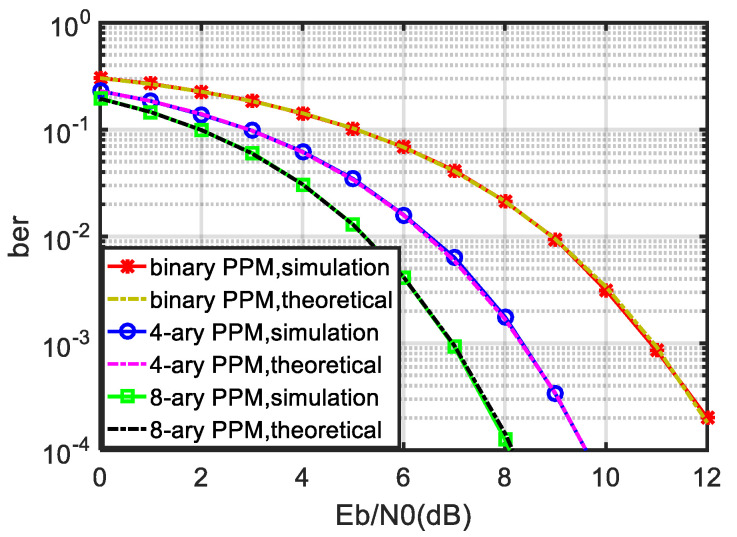
BER performance of noncoherent PPM optimal receiver.

Next, the best bit error performance of the noncoherent PPM receiver is numerically simulated, and the binary PPM, 4-ary PPM and 8-ary PPM are included for comparison. The value range of $E_{b}/N_{0}$ is 0–12 dB, and the BER is counted as $10^{-4}$. The simulation results are shown in Figure 7.

The theoretical curve in Figure 7 is calculated by the theoretical formula derived in Section 3.2. The simulation curve is the numerical simulation result. The simulation process first randomly generates the initial binary bit stream A, modulates it into a PPM signal, then sends it to AWGN channel and then restores it to binary bit stream B through the optimal receiver in this paper. We compare the similarities and differences between B and A and calculate the BER to obtain the simulation BER curve simulation.

It can be seen from Figure 7 that the numerical simulation results are in good agreement with the theoretical formula derived in Section 3.2, indicating that the noncoherent optimal receiver bit error theoretical formula derived in this paper can be applied to a *Q*-ary PPM system, including binary PPM. It can also be seen that the larger the *Q*, the better the BER performance of the noncoherent PPM receiver, which is also consistent with the fact that PPM corresponds to an orthogonal signal. By comparing Figure 6 with Figure 7, it can also be seen that the bit error performance of the optimal receiver of the noncoherent PPM is better than that of the suboptimal receiver at 2 dB.

### 4.3. Comparison between the Numerical and Theoretical Results

Table 1 and Table 2 illustrate the errors between the BER performance and the theoretical results of suboptimal receiver in noncoherent PPM system. The error is defined as

(24) $$error=\frac{∥P_{eb,num}-P_{eb,thy}∥}{P_{eb,thy}}\;,$$

where $P_{eb,num}$ represents the numerical result, $P_{eb,thy}$ denotes the theoretical result and ∥·∥ is the absolute value operator. The ensemble runs obtaining $P_{eb,num}$ is 100. In Table 1, each test uses $1\times 10^{3}$ PPM symbols, while the number of PPM symbols used for Table 2 is $1\times 10^{5}$. For convenience, we only analyzed the cases where the BER results are larger than $10^{-4}$. The theoretical results are derived based on the assumption that the PPM symbols are uniformly distributed in the possible time slots. However, the numerical simulation can not accurately meet the assumption. Thus, there are certain errors between the numerical results and the theoretical results. It can be seen from Table 1 and Table 2 that, as the number of PPM symbols increases, the deviation between the numerical results and the theoretical results tends to decrease. The reason for this is that the PPM symbols asymptotically obey the uniform distribution in the possible time slots when the number of PPM symbols increases.

Table 3 and Table 4 demonstrate the errors for optimal receiver in noncoherent PPM system. The results are similar to that of suboptimal receiver.

### 4.4. BER Performance in Rayleigh Fading Channel

The AWGN channel is a simple but useful model for the preliminary performance analysis of many communication systems. However, the real channel is more complex in many application scenarios in which the receivers need to overcome ISI [26] and MAI [27]. The performance analysis of some receivers is carried out in specific channel models [28]. The performance of the modulation scheme combining PPM with differential chaos shift keying modulation in Rayleigh fading channel is of considerable interest [29]. Therefore, the BER performance of the suboptimal receiver and the optimal receiver of noncoherent Q-ary PPM system in the Rayleigh fading channel is initially investigated through numerical simulation.

The received signal in multipath Rayleigh fading channel can be formulated as

(25) $$r\left(t\right)=\sum_{l=1}^{L}\alpha_{l}\delta \left(t-\tau_{l}\right)⊗s_{l}\left(t\right)+n_{l}\;,$$

where *L* is the number of paths, $\alpha_{l}$ and $\tau_{l}$ are the channel coefficient and the path delay of the path, and ⊗ denotes the convolution operator. $n_{l}$ is the AWGN with zero mean and variance $N_{0}/2$. The values of $E_{b}/N_{0}$ ranges from 0 dB to 12 dB. We consider the case where the BER is larger than $10^{-4}$. The width of a time slot is 5 ns. The other parameters are set as L=2, $\alpha_{l}=\left\{0,-2\right\}$ dB and $\tau_{l}=\left\{0,2\right\}$ ns.

**Figure 8:**
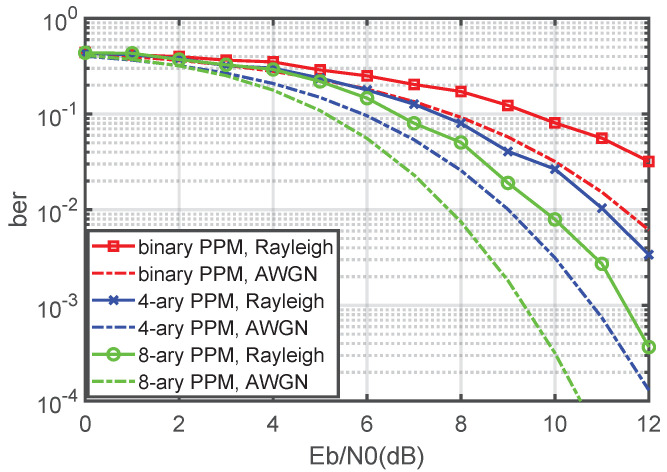
BER performance of a noncoherent Q-ary PPM suboptimal receiver in the Rayleigh fading channel and AWGN channel.

**Figure 9:**
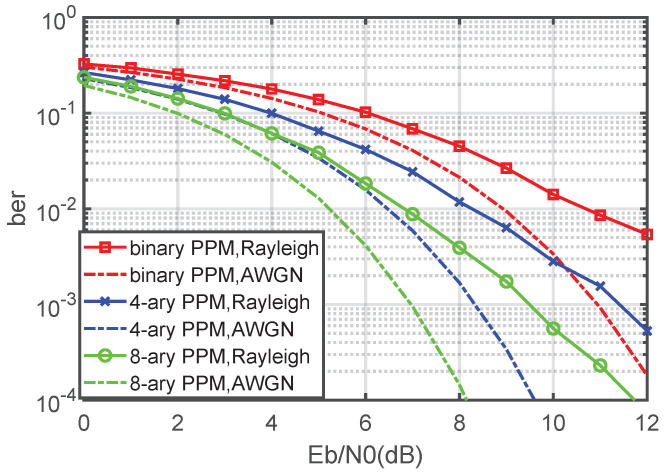
BER performance of a noncoherent Q-ary PPM optimal receiver in the Rayleigh fading channel and AWGN channel.

Figure 8 and Figure 9 show the BER results of the suboptimal receiver and the optimal receiver when the channel is Rayleigh and AWGN. Compared with the AWGN channel, the BER performance of the noncoherent Q-ary PPM system decreases in the Rayleigh fading channel. As the focus of this paper is the AWGN channel for which the receivers are designed, it is reasonable that the BER degradation occurs in the Rayleigh fading channel. An interesting research subject is studying the performance of a noncoherent Q-ary PPM system in a fading channel in more detail and improving the BER performance.

## 5. Conclusions

This paper introduces the PPM signal structure and introduces the decision method of a noncoherent PPM suboptimal receiver and optimal receiver. Of these, the suboptimal receiver needs to set a decision threshold and to make a decision by comparing the time slot envelope sampling value with the threshold in turn. The optimal receiver does not need to set a threshold but needs to compare the envelope sampling values of each time slot with each other and to determine the largest one as the transmitted symbol. The performance of the optimal receiver is about 2 dB better than that of the suboptimal receiver, but the suboptimal receiver is easier to implement. Therefore, it is necessary to comprehensively consider which receiver to choose in practical application.

In this study, the bit error theoretical formulas of a noncoherent PPM suboptimal receiver and optimal receiver in the AWGN channel were derived, respectively, and a numerical simulation was carried out. The numerical simulation results are in good agreement with the calculation results of the theoretical formula, which verifies the correctness of the error code theoretical formula, especially that the deduced error code theoretical formula is suitable for a *Q*-ary PPM system including binary PPM. The relationship between the threshold coefficient of the suboptimal receiver and the system error performance is given, which can be used to guide the selection of the threshold coefficient. In addition, the research results show that the larger the *Q*, the better the bit error performance of the suboptimal receiver and the optimal receiver of noncoherent PPM but the more difficult it is to realize. Therefore, it is necessary to comprehensively consider and select an appropriate *Q* value in practical applications.

## Figures and Tables

**Table 1:** The errors between the BER performance and the theoretical results of suboptimal receiver ($100\times 10^{3}$ PPM symbols).

$E_{b}/N_{0}$ (dB)	0	1	2	3	4	5	6	7	8	9	10	11	12
Binary (%)	2.1	3.6	3.1	0.7	3.1	4.5	0.3	1.2	0.3	3.9	1.3	2.8	0.4
4-ary (%)	0.4	1.7	1.4	3.8	0.3	2.7	2.5	1.8	1.5	1.3	1.4	6.8	7.6
8-ary (%)	4.8	0.3	0.5	4.1	3.1	5.1	0.3	3.0	3.6	1.8	20.7	/	/

**Table 2:** The errors between the BER performance and the theoretical results of suboptimal receiver ($100\times 10^{5}$ PPM symbols).

$E_{b}/N_{0}$ (dB)	0	1	2	3	4	5	6	7	8	9	10	11	12
Binary (%)	0.4	0.4	1.2	1.4	1.6	1.2	0.3	1.2	0.9	1.4	0.5	0.2	0.1
4-ary (%)	0.9	1.1	0.8	2.1	1.0	1.2	2.4	1.1	1.8	0.3	0.3	0.7	1.2
8-ary (%)	1.5	0.7	1.3	1.0	1.2	0.6	1.1	1.0	0.1	1.1	3.2	/	/

**Table 3:** The errors between the BER performance and the theoretical results of optimal receiver ($100\times 10^{3}$ PPM symbols).

$E_{b}/N_{0}$ (dB)	0	1	2	3	4	5	6	7	8	9	10	11	12
Binary (%)	1.6	0.9	2.2	1.1	1.5	2.3	2.7	1.3	3.5	3.7	1.5	2.3	5.4
4-ary (%)	2.5	3.3	0.8	1.9	2.5	2.6	0.9	1.5	6.1	9.3	/	/	/
8-ary (%)	2.3	1.5	2.3	0.7	2.5	1.7	3.5	4.6	17.9	/	/	/	/

**Table 4:** The errors between the BER performance and the theoretical results of optimal receiver ($100\times 10^{5}$ PPM symbols).

$E_{b}/N_{0}$ (dB)	0	1	2	3	4	5	6	7	8	9	10	11	12
Binary (%)	0.5	1.5	0.2	1.0	1.2	0.9	0.5	2.1	0.7	0.8	1.1	0.3	0.5
4-ary (%)	1.2	0.6	0.9	0.5	1.5	2.1	1.7	0.9	1.3	0.8	/	/	/
8-ary (%)	0.4	1.2	2.2	1.4	0.8	1.3	0.9	1.3	1.6	/	/	/	/

## Data Availability

Not applicable.
